# CUT&Tag and DiBioCUT&Tag enable investigation of the AT-rich epigenome of *Plasmodium falciparum* from low-input samples

**DOI:** 10.1016/j.crmeth.2025.101110

**Published:** 2025-07-16

**Authors:** Jonas Gockel, Gala Ramón-Zamorano, Jessica Kimmel, Tobias Spielmann, Richárd Bártfai

**Affiliations:** 1 Department of Molecular Biology, Radboud University, 6525GA Nijmegen, the Netherlands; 2 Pathogen Section, Bernhard Nocht Institute for Tropical Medicine, 20359 Hamburg, Germany

**Keywords:** *Plasmodium falciparum*, epigenomic profiling, CUT&Tag, DiBioCUT&Tag, heterochromatin, CenH3

## Abstract

Phenotypic variation between malaria parasites is a major contributor to the pathogen’s success, facilitated by heritable yet dynamic changes in (hetero)chromatin structure. Currently, the chromatin landscape is mostly profiled by chromatin immunoprecipitation sequencing (ChIP-seq), which has several drawbacks: (1) GC-content-related artifacts, (2) substantial material requirement, and (3) a labor-intensive protocol. To overcome these limitations, we adapted cleavage under targets and tagmentation (CUT&Tag) to *Plasmodium falciparum*. Despite the AT richness of the genome, CUT&Tag results in reproducible heterochromatin profiles concordant with ChIP-seq data while using as little as 10,000 nuclei or crude parasite isolates. We also developed DiBioCUT&Tag, a method utilizing dimerization-induced recruitment of biotin ligase for proximity labeling of core chromatin components during the binding of regulatory proteins followed by anti-biotin CUT&Tag. These methods hence provide substantially improved means for genome-wide profiling of chromatin-associated proteins from low-input samples in the malaria parasite and potentially beyond.

## Highlights

• Reliable heterochromatin profiling of the AT-rich *P. falciparum* genome by CUT&Tag
• Low-input CUT&Tag requires as few as 10,000 nuclei/parasites
• CUT&Tag with prior proximity biotinylation improves genome-wide profiling signals

## Motivation

Epigenetic regulation enables adaptation and survival of the deadly malaria parasite *P. falciparum*. However, genome-wide profiling of different epigenetic states requires significant amounts of input material, severely limiting the investigation of sparse sample types (e.g., mosquito stages or field isolates). Furthermore, most methods are prone to GC-content-related artifacts, which are exaggerated due to the exceptionally high AT content in this organism.

To overcome these limitations, we adapted CUT&Tag to *P. falciparum* for standard and low-input samples, as well as utilized crude/frozen parasites as input material. Furthermore, we developed DiBioCUT&Tag, coupling dimerization-induced biotinylation to respective targets to carry out anti-biotin CUT&Tag.

## Graphical abstract

**Figure:**
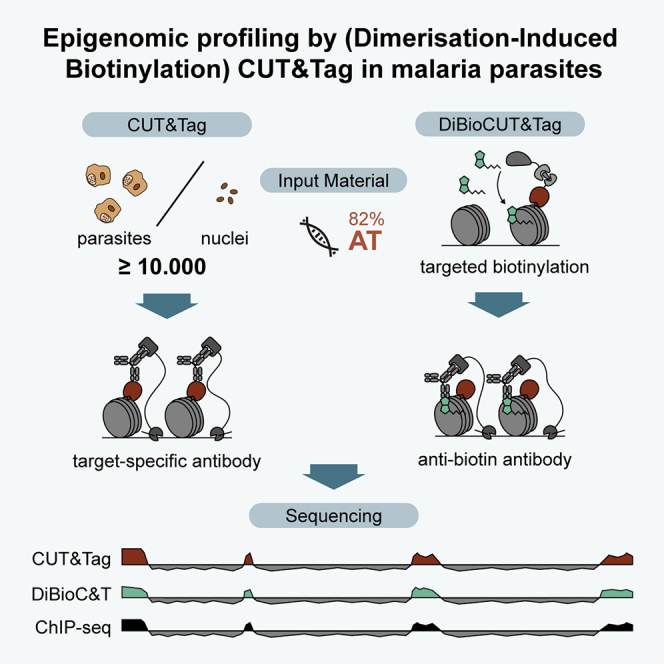

## Teaser

Gockel et al. present a version of the epigenomic profiling technique CUT&Tag adapted to the AT-rich genome of the deadly parasite *Plasmodium falciparum*. This low-input epigenomic profiling protocol enables studies of scarce sample types such as field isolates. Technical advances in coupling CUT&Tag with prior proximity biotinylation further increase the signal for (temporal) chromatin-associated factors.

## Introduction

Epigenetic regulatory mechanisms influence cellular differentiation by activating or repressing the expression of genes that impact cellular fate. Furthermore, they enable short-term adaptability of organisms next to longer-term adaptation due to DNA sequence alterations. Epigenetic regulation is mainly achieved by posttranslational modification of histones and consequent alteration of chromatin structure and accessibility via the effector proteins. A classical and conserved example is the formation of transcriptionally inactive heterochromatin via deposition of methyl marks on lysine 9 of histone H3 (H3K9me3) and consequent binding and oligomerization of heterochromatin protein 1 (HP1).^1^^,^^2^^,^^3^^,^^4^^,^^5^

While epigenetic regulation is extensively studied in the context of vertebrate development, it is much less understood in eukaryotic pathogens like *Plasmodium falciparum*, the causative agent of malaria. Nonetheless, accumulating evidence suggests that developmental transitions in the complex life cycle of these parasites, transmitting between human and mosquito hosts, are largely dependent on epigenetic regulation.^6^^,^^7^^,^^8^ Furthermore, heterochromatin-mediated silencing at the chromosome ends and at some chromosome internal islands contributes to drug resistance, host-specific adaptation of invasion ligands, and evasion of the immune system via antigenic variation.^9^^,^^10^^,^^11^^,^^12^^,^^13^ Finally, given its essential function, epigenetic regulation is a potent target for drug development.^14^

Previous studies investigating genome-wide distribution of epigenetic marks and chromatin-associated proteins in malaria parasites almost exclusively employed chromatin immunoprecipitation (ChIP) followed by next-generation sequencing.^7^^,^^15^^,^^16^ ChIP relies on affinity-based purification of formaldehyde-crosslinked and sonicated chromatin fragments containing a transcription factor or epigenetic marks of interest targeted with a specific antibody. While ChIP sequencing (ChIP-seq) can provide reliable results, it has some limitations^17^: (1) given the inefficiency of immunoprecipitation, it requires millions of cells as input material, (2) chromatin fragmentation by sonication is batch sensitive, and (3) formaldehyde fixation can influence antibody binding, cannot efficiently capture transient interactions, and leads to biases for GC-rich sequences. These shortcomings are further exacerbated in *P. falciparum* owing to amplification and sequencing biases during analysis of its extremely AT-rich genome.^18^^,^^19^^,^^20^ Collectively, while ChIP-seq has been instrumental in the initial exploration of the *Plasmodium* epigenome, it does not allow for the investigation of any samples with limited availability of material, such as field isolates and mosquito- and liver-stage parasites, let alone individual parasites.

More modern epigenetic profiling techniques developed for model eukaryotes, such as CUT&RUN^21^ or cleavage under targets and tagmentation (CUT&Tag),^22^ are aiming to minimize these shortcomings of ChIP-seq. To perform a CUT&Tag experiment, cells or isolated nuclei/permeabilized cells are bound to concanavalin A beads as a platform for subsequent incubation and wash steps. Epigenetic marks of interest are targeted with a specific antibody, which is incubated with the nuclei, followed by a secondary antibody to amplify the signal. These antibodies then direct a proteinA-Tn5 transposase fusion protein to a specific epigenetic feature, at which loci tagmentation with sequencing adapters is induced by the addition of Mg^2+^ ions. All these processes occur within the nuclei, which are then lysed and their DNA extracted. Libraries are prepared by PCR amplification of short fragments with primers complementary to the integrated adapter sequences, which, following size selection, are sequenced on a compatible next-generation sequencing platform. Therefore, as opposed to ChIP-seq, chromatin is not randomly fragmented by mechanical force, but instead, fragments are generated by the integration of sequencing adapters, which are then specifically amplified. CUT&Tag is more efficient than ChIP-seq with a significantly reduced background signal and can therefore be performed on limited input material and has even been used for single-cell epigenetic profiling of histone marks.^22^^,^^23^^,^^24^

In this work, we adapted and optimized the epigenetic profiling technique CUT&Tag to the extremely AT-rich (average 82% AT) *P. falciparum* parasites. Our protocol can reliably and consistently profile the heterochromatin landscape on both H3K9me3 and HP1 antibody targets. Furthermore, we show that CUT&Tag is suitable for both low-input material down to 10,000 nuclei as well as crude whole parasite isolations. Importantly, we developed an approach utilizing dimerization-induced recruitment of a biotin ligase (miniTurbo) via a chromatin-associated protein (HP1 and CenH3) and CUT&Tag profiling of the corresponding chromatin regions by an α-biotin antibody. This approach (named DiBioCUT&Tag) is not affected by the loss of the transient chromatin interaction due to high salt washes during the standard CUT&Tag protocol and hence can potentially be applied to profile transient chromatin interactors.

## Results and discussion

### CUT&Tag enables efficient heterochromatin profiling in the AT-rich genome of *P. falciparum*

**Figure 1:**
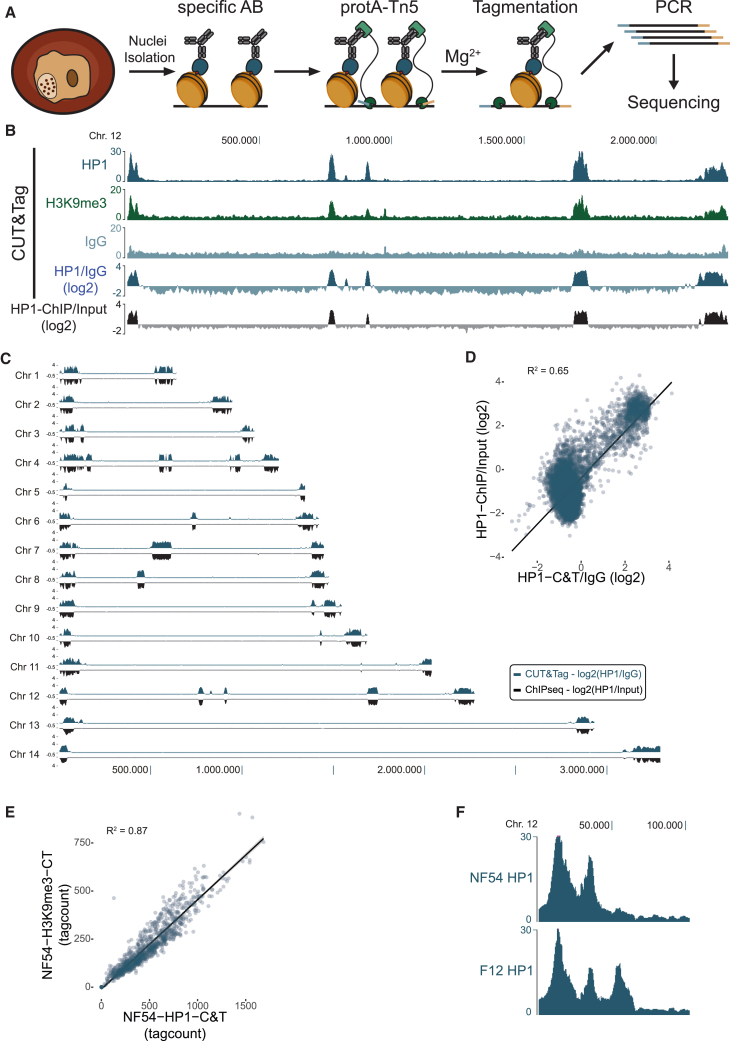
CUT&Tag of HP1 and H3K9me3 in *Plasmodium falciparum* provides accurate means to genome-wide heterochromatin profiling (A) The main steps of CUT&Tag. Nuclei are isolated from parasites and incubated with a specific antibody (AB) against the target of interest. This AB directs a proteinA-Tn5 transposase fusion protein to specific chromatin regions. Transposition of sequencing adaptors (tagmentation) is induced by the addition of Mg^2+^ ions, and the resulting DNA fragments are amplified by PCR and then subjected to massive parallel sequencing. (B) Chromosome-wide CUT&Tag profiles for HP1 (blue), H3K9me3 (green), and control IgG (normalized read count or log2 ratio tracks, light blue) (100,000 nuclei input material) as well as an HP1 ChIP-seq (ChIP/input, black). Log2 ratio tracks have been calculated in 500-bp windows. (C) Genome-wide profiles of background-corrected CUT&Tag (blue) and ChIP-seq (black) tracks from (B). (D) Scatterplot displaying genome-wide correlation between HP1 ChIP-seq and CUT&Tag log2 ratio tracks in 2,000-bp windows. Note that the two different clusters represent euchromatic and heterochromatic regions, respectively. (E) Scatterplot displaying correlation between HP1 and H3K9me3 CUT&Tag read counts in 2,000-bp windows genome wide. Euchromatic regions (tag counts < 150) are not shown. (F) HP1 CUT&Tag normalized tag count tracks at the distal end of chromosome 12 in two different *P. falciparum* strains (NF54 and F12), highlighting strain-specific differences in heterochromatin occupancy as previously described by Fraschka et al.^7^

To test the utility of CUT&Tag for the extremely AT-rich genome of *P. falciparum* parasites, we isolated and permeabilized nuclei from trophozoite and schizont stages. We followed the basic principle of the technique described by Kaya-Okur et al.^22^^,^^25^ (Figure 1A), optimizing both nuclei isolation, permeabilization, and PCR protocols for *P. falciparum* cells (see STAR Methods for details). Genome-wide occupancy profiles of both HP1 and H3K9me3 CUT&Tag showed a heterochromatin landscape very similar to ChIP-seq profiles (Figures 1B and S1A). Signal-to-noise ratio comparisons between ChIP-seq and CUT&Tag show improvement in CUT&Tag samples (6.2 and 13.1, respectively). To test any technical biases toward GC-rich heterochromatic or other genomic regions influencing CUT&Tag results, we also performed CUT&Tag with a non-specific immunoglobulin (IgG) antibody. The resulting genome occupancy tracks showed an almost flat background profile (Figure 1B, light blue), indicating the lack of substantial biases (Figure S1B). Utilizing the IgG read count as the background, we also corrected the HP1 CUT&Tag read counts into a log2 ratio track, providing an even more accurate measure of heterochromatin occupancy (Figures 1B and 1C). We next performed a genome-wide quantitative analysis in 2,000 bp windows throughout the genome to compare HP1 CUT&Tag and ChIP-seq (Figure 1D), as well as HP1 and H3K9me3 CUT&Tag datasets (Figure 1E), both of which showed a positive correlation (R^2^ = 0.65 and R^2^ = 0.87, respectively), demonstrating accuracy and reliability of the obtained heterochromatin profiles. Importantly, HP1 CUT&Tag, similar to ChIP-seq, enables clear separation of heterochromatin and euchromatic regions indicated by the two clusters visible in Figure 1D. H3K9me3 CUT&Tag provided a somewhat lower signal-to-noise ratio as compared to previous ChIP-seq experiments (Figure S1C). To verify the sensitivity of our protocol, we examined heterochromatin differences between two different *P. falciparum* strains, previously reported by Fraschka et al*.*^7^ We observed the expected strain-specific differences between NF54 and 3D7 (F12), including an extension of the heterochromatic domain on the proximal end of chromosome 12 (Figure 1F). These observations demonstrate the utility and accuracy of CUT&Tag for heterochromatin profiling even on an AT-rich genome such as that of the malaria parasite, *P. falciparum*.

### CUT&Tag can be scaled down to 10,000 nuclei without losing heterochromatin calling efficiency

**Figure 2:**
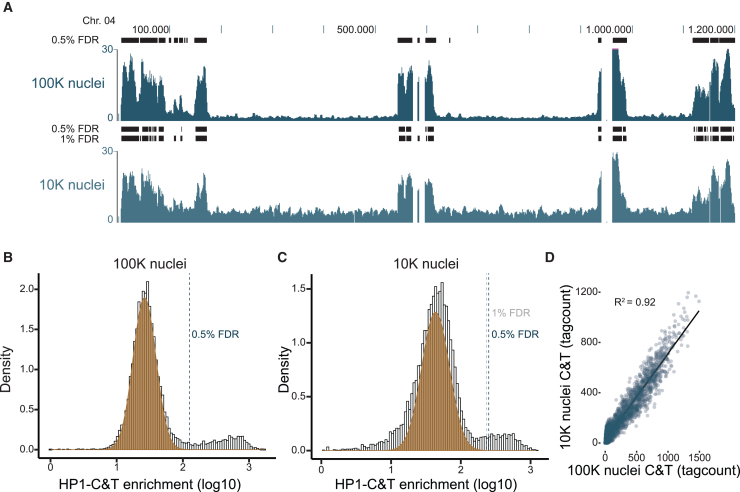
CUT&Tag enables identification of heterochromatic regions also from low-input samples (A) Chromosome-wide CUT&Tag profiles for HP1 using standard input (100,000 nuclei, dark blue) as well as low input (10,000 nuclei, light blue). Black bars represent 2,000-bp windows with signal significantly higher than background (as determined in B and C with false discovery rates [FDRs] of 0.5% and 1% for 10,000 nuclei). For another low-input CUT&Tag experiment, see Figures S1D and S2E. (B) Histogram depicting read count distribution in 2,000-bp windows for HP1 CUT&Tag using 100,000 nuclei. Normal distribution model (orange) for background signal and 0.5% FDR cutoff for heterochromatin calling (dotted line) are indicated. (C) Histogram depicting read count distribution in 2,000-bp windows for HP1 CUT&Tag using 10,000 nuclei. Normal distribution model (orange) for background signal and 0.5% FDR cutoff for heterochromatin calling (black dotted line) and 1% FDR cutoff for 10,000 nuclei (gray dotted line) are indicated. (D) Scatterplot displaying correlation between 100,000 and 10,000 nuclei samples in 2,000-bp windows genome wide.

One of the major advantages of CUT&Tag over ChIP-seq is its potential to scale down input material and profile heterochromatin in sparse sample types. To test whether CUT&Tag is applicable for these low-input conditions, we performed HP1 CUT&Tag on as little as 10,000 nuclei. Genome-wide occupancy profiles between 100,000 and 10,000 nuclei showed very similar heterochromatin landscapes (see Figures 2A and S1D for independent experiments with 50,000 and 10,000 nuclei, respectively), with only a slight decrease in the signal-to-noise ratio in the 10,000 nuclei sample. The background and signal, however, remained still clearly distinguishable (Figures 2B and 2C) and enabled efficient heterochromatin calling (Figure 2A, black boxes) with 0.5%–1% false discovery rate (FDR) based on a modeled normal distribution of background signal (Figures 2B and 2C, orange area). Furthermore, genome-wide quantitative analysis showed a high positive correlation (R^2^ = 0.92) between low- and regular-input reactions (Figure 2D). Therefore, CUT&Tag enables heterochromatin profiling from as few as 10,000, and possibly even fewer, nuclei.

### Nuclei isolation is not essential for CUT&Tag, enabling more efficient sample processing from intact parasites

**Figure 3:**
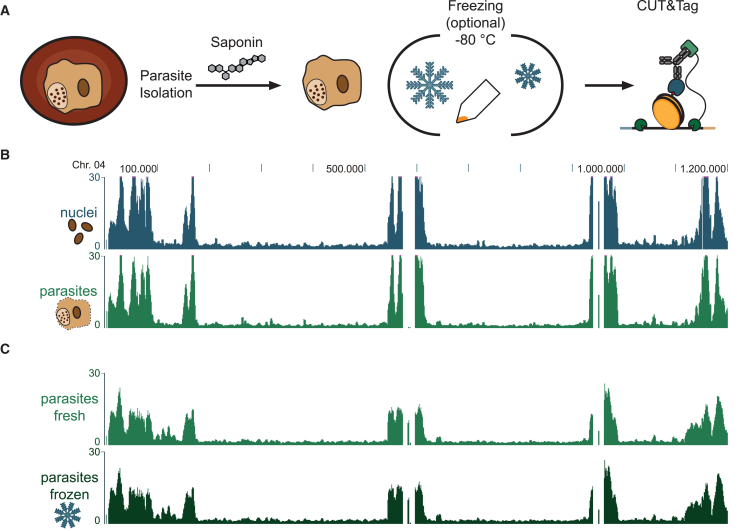
CUT&Tag can also be performed on frozen and/or intact parasites without isolation of nuclei (A) Schematics of parasite isolation for CUT&Tag. Parasites are isolated by saponin-mediated lysis of the red blood cell and can be either used directly for CUT&Tag or snap frozen in liquid nitrogen and stored at −80°C. (B) Chromosome-wide HP1 CUT&Tag profiles using 1 million fresh isolated nuclei (blue) and 1 million parasites (green). (C) Chromosome-wide HP1 CUT&Tag profiles using 100,000 isolated parasites directly (fresh, light green) or after freezing (frozen, dark green).

The process of isolating nuclei is both laborious and leads to substantial sample loss. Furthermore, direct processing of the samples for CUT&Tag (e.g., in endemic settings) is not always possible, and hence storage is desirable. To further optimize our CUT&Tag protocol, we pursued the use of intact, isolated parasites (either directly or after frozen storage) as an input material instead of nuclei (Figure 3A). Parasites were released from infected cells by saponin-mediated lysis of the red blood cells and were then either used directly as CUT&Tag input or snap frozen for storage (Figure 3A). Importantly, neither skipping the nuclei isolation step (Figures 3B, S2A, and S2C) nor freezing (Figures 3C, S2B, and S2D) impaired heterochromatin landscape profiling. Furthermore, performing low-input CUT&Tag down to 10,000 parasites still gives reliable heterochromatin profiles (Figure S2E). These results show that we can generate reliable results when using frozen parasite isolates for CUT&Tag experiments and in turn minimize sample preparation time and improve the efficiency and flexibility of these experiments.

### DiBioCUT&Tag is a dimerization-induced, proximity-labeling-based approach for epigenetic profiling

**Figure 4:**
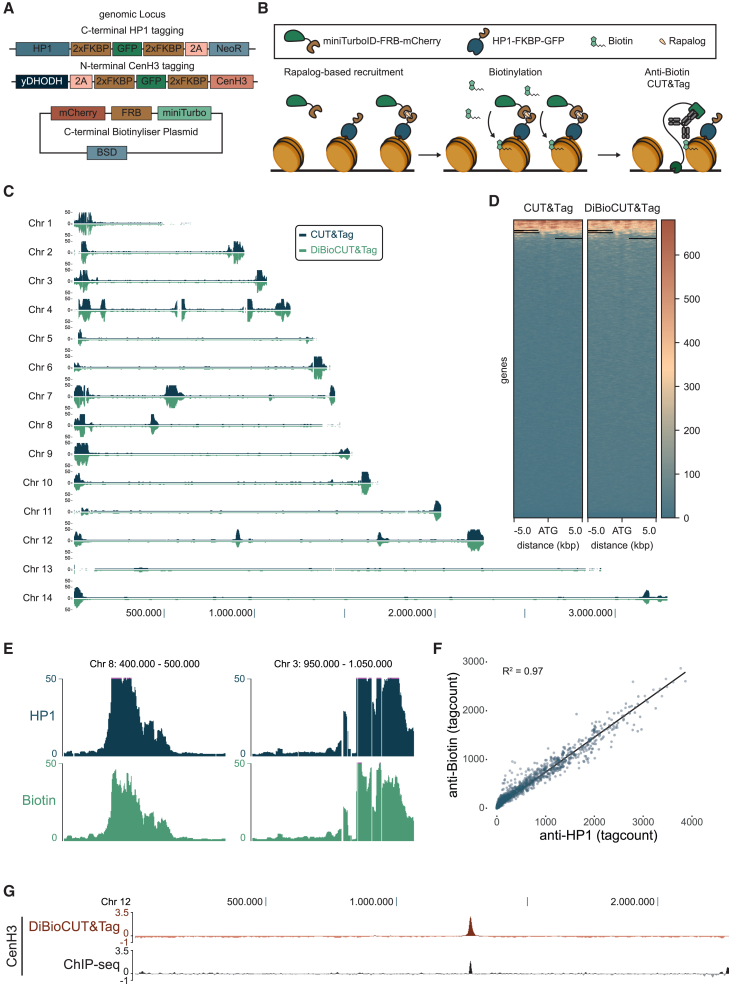
BioCUT&Tag is a proximity-labeling-based amplification for epigenetic profiling (A) Drawing depicting endogenous tagging of the HP1 locus with 2xFKBP-GFP^26^ and an episomal plasmid carrying mCherry-FRB-miniTURBO construct. (B) Schematic of BioCUT&Tag. The biotinylizer, miniTurbo-FRB-mCherry, is recruited to the target protein, HP1-FKBP-GFP, by addition of rapalog. Upon the addition of biotin, chromatin-associated proteins in the vicinity of HP1 are biotinylated. These biotin molecules are then targeted with a specific anti-biotin antibody in the subsequent CUT&Tag reaction. (C) Genome-wide read occupancy tracks from HP1 (blue) and biotin (green) CUT&Tag. (D) Heatmap depicting genome-wide signal of HP1 CUT&Tag and DiBioCUT&Tag in relation to the ATG of all genes. (E) Read occupancy tracks from HP1 (blue) and biotin (green) CUT&Tag at two specific locations. (F) Scatterplot displaying genome-wide correlation between HP1 and Biotin CUT&Tag read counts in 2,000-bp windows genome wide. (G) Log2 ratio tracks from CenH3 DiBioCUT&Tag (orange) and cenH3 ChIP-seq (black). CenH3 ChIP-seq is from Hoeijmakers et al.^27^

One of the main limitations of CUT&Tag is the difficulty of applying it to the profiling of temporarily chromatin-associated factors (e.g., transcription factors, chromatin-modifying enzymes, or reader proteins). Strong salt washes (specifically 300 mM NaCl) can dissociate weakly interacting factors from chromatin and are therefore potentially not profiled. In order to overcome this limitation, we included an additional step of conditionally biotinylating strongly chromatin-associated proteins (e.g., histones) in the vicinity of the target protein and performing CUT&Tag with an α-biotin antibody (Figure 4B). This, in principle, should lead to the accumulation of the signal over time and the amplification of weak signals. As a proof of principle, we use a parasite line in which HP1 is FKBP tagged^26^ and transfected it with a plasmid carrying an FRB-miniTurbo biotin ligase fusion protein (Figure 4A). Upon the addition of rapalog, FKBP and FRB dimerize, and miniTurbo is recruited to HP1-occupied regions, where resident histones are then biotinylated (Figure 4B). Targeting these biotinylation events with an α-biotin antibody in a CUT&Tag reaction led to reliable heterochromatin profiling (Figure 4C), with almost identical signals for HP1/biotin CUT&Tag (Figures 4D and 4E) and a high correlation between the CUT&Tag and DiBioCUT&Tag profiles (Figure 4F, R^2^ = 0.97). To demonstrate the utility of DiBioCUT&Tag for a different epigenetic feature, we also profiled the centromeric histone variant CenH3. This shows a very clear and specific signal similar to previous ChIP-seq experiments^27^ when using the minus rapalog sample as a control (Figures 4G and S3A). These experiments provide proof of principle that DiBioCUT&Tag can be used for the profiling of heterochromatin and can be expanded to other chromatin-associated proteins (with a caveat described in the limitations of the study section).

## Discussion

In this study, we show that CUT&Tag is a robust technique to reliably profile heterochromatic regions even in the AT-rich genome of *P. falciparum*. Limiting input material to 10,000 nuclei or parasites still led to reproducible results with clear separation of euchromatic and heterochromatic regions. Furthermore, fresh and frozen intact parasite isolations provided very similar and reliable heterochromatin readouts and hence can be used to minimize sample loss during processing. Avoiding purification of nuclei and the option to store frozen samples also increases the potential range of sample sources (clinical or endemic settings) and simplifies as well as reduces the time of the protocol. CUT&RUN^28^^,^^29^ and CUT&Tag^30^ have recently been used for the profiling of histone modifications in *P. falciparum* parasites. However, their utility to low-input samples was not addressed. Accordingly, the developments described here open avenues toward the investigation of scarce sample types, such as field isolates or mosquito and liver stages of parasite development. Furthermore, CUT&Tag has been proven to be applicable at the single-cell level,^22^^,^^23^^,^^24^ which, in the case of *Plasmodia*, has the potential to revolutionize the exploration of epigenetic variation between individual parasites that may underly developmental decisions or virulence factor expression on the individual cell level. However, based on our experiments (Figure 2), we predict a further decline in the signal-to-noise ratio, which results in very sparse data at the single-parasite level. Therefore, single haploid parasite CUT&Tag will most likely only be applicable to stable and broadly distributed epigenetic features, such as heterochromatin. However, DiBioCUT&Tag might sufficiently amplify signals of other chromatin-bound factors to enable their profiling at the single-cell level.

Our DiBioCUT&Tag data display a different heterochromatin footprint than our HP1 CUT&Tag data in NF54 due to the different maternal strain used in the generation of the transgenic line used for DiBioCUT&Tag. Furthermore, clonal variance in heterochromatin occupancy is well reported^7^ and can explain some slight phenotypic variation between our datasets.

Despite multiple advantages, CUT&Tag, similar to CUT&RUN and ChIP-seq, depends on the availability of specific and compatible antibodies. Furthermore, the high salt concentrations necessary to quench unspecific tagmentation events lead to inefficient capturing of transient interactions, such as the binding of transcription factors and effector proteins.^25^ To overcome these limitations, we developed an approach where strongly chromatin-associated proteins (e.g., histones) in the vicinity of the binding site are biotinylated and later profiled by anti-biotin CUT&Tag (DiBioCUT&Tag). CUT&Tag of the biotinylated chromatin enables the use of a standard α-biotin antibody^31^ or even a fusion of streptavidin with Tn5 transposase (which would enable taking advantage of the exquisite properties of the streptavidin-biotin interaction) but requires genetic modification of the target proteins.

Tn5-based integration of sequencing adapters has a bias toward GC-rich regions and can, together with more favorable PCR conditions on high-GC regions, lead to weakened signal-to-noise ratios at, for example, telomeres. However, these biases can be corrected with an IgG or other untargeted antibody control.

In summary, we present CUT&Tag and DiBioCUT&Tag as a reliable, cost- and time-effective alternative to ChIP-seq epigenetic profiling in *P. falciparum* (and potentially other organisms, such as *Dictyostelium discoideum*, with a very high AT content). These advances will be imperative to analyze sparse sample types during the parasite life cycle, deepening our understanding of this deadly parasite as well as potentially revealing new epigenetic drug targets.

### Limitations of the study

When profiling chromatin-associated proteins with DiBioCUT&Tag, special attention needs to be directed to the unique background signal of untethered biotin ligase in open chromatin regions. We learned this while attempting to profile the genome-wide binding of the BDP5 chromatin reader protein (Figure S4A). Specifically, we profiled “biotin footprints” left behind by BDP5-FKBP + FRB-TurboID dimers at two different parasite stages (trophozoites, 24–32 h post-infection [hpi] and schizonts, 32–40 hpi) following short rapalog and biotin treatment. DiBioCUT&Tag gave rise to clear local enrichments and distinct peak profiles in both samples (Figure S4A), which could not be observed in the generally flat and noisy “profile” obtained in the absence of both rapalog and biotin. Bioinformatic analysis of these profiles highlighted a clear enrichment of the DiBioCUT&Tag signal upstream of ATG in a stage-specific manner (Figures S4B–S4D) compared to CUT&Tag IgG controls (-biotin, -rapalog profiles were too sparse for this purpose). To our surprise, however, in the absence of rapalog and the addition of biotin, we did already observe a profile very reminiscent of the rapalog/biotin-plus profiles (Figure S4A). These data could, in principle, be explained by either recruitment of the miniTurbo enzyme in the absence of rapalog or by background biotinylation of open chromatin regions by “free-floating” miniTurbo. To differentiate between these two options, we used a parasite line carrying an episomal FRB-miniTurbo expression plasmid in the absence of any FKBP-tagged proteins and performed DiBioCUT&Tag on these parasites in the absence or presence of rapalog and/or biotin. This experiment clearly indicated that free-floating miniTurbo preferentially biotinylates open chromatin regions and results in an ATAC-seq-like background in DiBioCUT&Tag experiments (Figure S4E). Notably, this signal does not originate from the CUT&Tag reaction itself, as shown by biotin-minus and anti-GFP controls (Figures S4A and S4E). Furthermore, this background was also negligible in the HP1 DiBioCut&Tag experiment (Figure 4C). Therefore, caution should be applied when using DiBioCUT&Tag to profile proteins binding to open chromatin regions, as it may be difficult, though not necessarily impossible, to differentiate real signals from the background. For these cases, direct coupling of the biotin ligase to the target protein will eliminate any free-floating biotin ligase and likely lead to more robust epigenomic profiling with an optimal signal-to-noise ratio.

## Resource availability

### Lead contact

Requests for further information and resources should be directed to and will be fulfilled by the lead contact, Richárd Bártfai (r.bartfai@science.ru.nl).

### Materials availability

Generated plasmids/cell lines are available from the lead contact upon request.

### Data and code availability

• All raw and processed sequencing data have been submitted to Gene Expression Omnibus (GEO) under reference number GEO: GSE270104.
• Code used for CUT&Tag data processing and visualization is available at GitHub (https://github.com/bartfai-lab/DiBio-CUTnTag-Analysis) and has been uploaded to Zenodo (10.5281/zenodo.15638929).
• Any additional information required to reanalyze the data reported in this work paper is available from the lead contact upon request.

## Acknowledgments

We are grateful to Till Voss (Swiss TPH, Basel) for sharing the α-PfHP1 antibody with us and for his advice throughout this project. We want to thank Andres Guillen for generating the mCherry-FRN-miniTurbo parasite line. J.G. and R.B. have received funding from the EU’s Horizon 2020 research and innovation programme (Cell2Cell ITN) under the Marie Skłodowska-Curie grant agreement number 860875. This work was further supported by a Leibniz Collaborative Excellence Grant (MalNucFunc; K328/2020 to T.S. and R.B.).

## Author contributions

Conceptualization, J.G. and R.B.; investigation, J.G., G.R.-Z., and J.K.; methodology, J.G.; formal analysis, J.G.; visualization, J.G.; data curation, J.G.; writing – original draft, J.G. and R.B.; writing – review & editing, G.R.-Z. and T.S.; resources, G.R.-Z., J.K., and T.S.; funding acquisition, T.S. and R.B.; supervision, R.B.

## Declaration of interests

The authors declare no competing interests.

## STAR★Methods

### Key resources table

**Table:** 

REAGENT or RESOURCE	SOURCE	IDENTIFIER
**Antibodies**		

Rabbit polyclonal anti-PfHP1	Brancucci et al.^11^	N/A
Rabbit polyclonal anti-H3K9me3	abcam	Cat#8898; RRID: AB_306848
Rabbit polyclonal anti-H3K9me3	Diagenode	Cat# C15410193; RRID: AB_2616044
Rabbit monoclonal anti-Biotin	Cell Signaling Technology	Cat#5597; RRID:AB_10828011
Mouse monoclonal anti-GFP	Roche	Cat#11814460001; RRID: AB_390913
Rabbit normal IgG control antibody	Millipore	Cat#12–370; RRID: AB_145841
Guinea pig polyclonal anti-Rabbit IgG	Antibodies-Online	Cat#ABIN101961; RRID: AB_10775589
Goat mixed monoclonal anti-Mouse IgG	EpiCypher	Cat#13–0048; RRID:AB_3676529

**Chemicals, peptides, and recombinant proteins**		

Formaldehyde 36.5–38%	Sigma-Aldrich	Cat#F8775
cOmplete™, EDTA-free Protease Inhibitor Cocktail	Roche	Cat#04693132001
BioMag®Plus Concanavalin A	Bangs Laboratories	Cat#BP531
CUTANA™ pAG-Tn5 for CUT&Tag	EpiCypher	Cat#15-1017

**Critical commercial assays**		

KAPA HiFi PCR Kit	Roche	Cat#07958838001
AMPure XP Reagent	Beckman Coulter	Cat# A63882
Agilent High Sensitivity DNA Kit	Agilent	Cat#5067-4626

**Deposited data**		

Raw and analyzed data	This Paper	GEO: GSE270104
NF54 HP1 ChIP-seq	Fraschka et al.^7^	GEO: GSE102695
NF54 H3K9me3 ChIP-seq	Michel-Todo et al.^13^	GEO: GSE208561
CenH3 ChIP-seq	Hoeijmakers et al.^27^	GEO: GSE270104

**Experimental models: Organisms/strains**		

Parasite Strain: *Plasmodium falciparum* F12	Alano et al.^32^	N/A
Parasite Strain: *Plasmodium falciparum* NF54	Delemarre and van der Kaay, 1979^33^	N/A
Parasite Strain: *Plasmodium falciparum* 3D7 HP1-2xFKBP-GFP-2A-NeoR/mCherry-FRB-miniTurbo	Birnbaum et al.^26^	N/A
Parasite Strain: *Plasmodium falciparum* 3D7 BDP5-2xFKBP-GFP-2A-NeoR/mCherry-FRB-miniTurbo	Hoeijmakers et al.^34^	N/A
Parasite Strain: *Plasmodium falciparum* 3D7 yDHODH-2A-GFP-cenH3/mCherry-FRB-miniTurbo	This Paper	N/A
Parasite Strain: *Plasmodium falciparum* 3D7 mCherry-FRB-miniTurbo only	This Paper	N/A

**Software and algorithms**		

Bowtie2 (v2.5.2)	Langmead et al.^35^^,^^36^	https://bowtie-bio.sourceforge.net/bowtie2/index.shtml
Picard (v3.1.0)	Broad Institute^37^	https://broadinstitute.github.io/picard/
Samtools (v1.18)	Li et al.^38^	
Deeptools (v3.5.4)	Ramírez et al.^39^	https://deeptools.readthedocs.io/en/develop/content/about.html
Bedtools (v2.31.0)	Quinlan and Hall^40^	https://bedtools.readthedocs.io/en/latest/index.html
Macs2 (v2.2.9.1)	Zhang et al. version 2^41^	https://pypi.org/project/MACS2/
R (v4.3.1)	R	https://www.r-project.org/
ggplot2 (v3.4.4)	Wickham et al.^42^	https://ggplot2.tidyverse.org/
Mixtools (v2.0.0.1)	Benaglia et al.^43^	https://cran.r-project.org/web/packages/mixtools/index.html
UCSC genome browser	UCSC genome browser^44^	http://genome.ucsc.edu
Analysis scripts used and documentation	This Paper	https://github.com/bartfai-lab/DiBio-CUTnTag-Analysis https://doi.org/10.5281/zenodo.15638929

### Experimental model and study participant details

#### *P. falciparum* cell culture

*P. falciparum* intraerythrocytic parasites were cultured at 37°C under low oxygen conditions (3% O_2_, 4% CO_2_ and 93% N_2_) in human red blood cells at 5% hematocrit in RPMI 1640 medium supplemented with 0.2% NaHCO_3_ and 10% human serum. Wild type parasites were grown in the absence of antibiotics. For DiBioCUT&Tag, the HP1-2xFKBP-GFP-2xFKBP-2A-NeoR/mCherry-FRB-miniTurbo, BDP5-2xFKBP-GFP-2xFKBP-2A-NeoR/mCherry-FRB-miniTurbo and mCherry-FRB-miniTurbo only strains were cultured in the presence of Gentamicin G-418 sulfate (400 μg/mL, Invitrogen, ant-gn-5) and Blasticidin (0.4 μg/mL, Invitrogen, ant-bl) in modified RPMI with L-glutamine and without biotin and phenol red (US biological life sciences, R9002-01) supplemented with 200 μM Hypoxyxanthine (Merck, H9377) and 0.5% AlbuMAX II (Gibco, 11021037). yDHODH-2A-2xFKBP-GFP-2xFKBP-cenH3/mCherry-FRB-miniTurbo parasites were cultured in medium containing DSM1 (0.9 μM, Sigma-Aldrich) and Blasticidin (0.4 μg/mL, Invitrogen, ant-bl). In order to achieve synchronicity, cultures were subjected to sorbitol-based lysis of remodeled RBCs [25]. Namely, infected RBCs were resuspended in 7 volumes of 5% Sorbitol and incubated at 37°C for 10 min. RBCs were washed once with RPMI 1640 medium supplemented with 0.2% NaHCO_3_ and 10% human serum and were cultured further under standard culture conditions. For biotinylation experiments, the medium was supplemented with 250 nM Rapalog (Clontech) 1h before harvest and 50 μm Biotin (Invitrogen, B20656) 30 min before harvest.

### Method details

#### Genetic modification of *P. falciparum* parasites

Integrated lines were obtained from previous studies. The generation of HP1-tagged line was described in Birnbaum et al.,^26^ whereas BDP5-tagged line was characterized in Hoeijmakers et al..^34^ N-terminal tagging of CENH3 was achieved using the SLI system.^26^ The gene’s 5′ homology region was cloned into the plasmid pSLI-N-sandwich-loxP between NotI and PmeI restriction sites, while containing a recodonized CENH3-coding sequence between AvrII and StuI restriction sites, using Gibson assembly.^45^ This line was maintained under DSM1 selection to ensure genomic integration. A correct integration was verified by PCR on gDNA with the primers shown in Figures S3C and S3D.^26^

The sequence encoding miniTurbo^46^ was a kind gift from the Gilberger lab. To produce the plasmid mCherry-FRB-miniTurbo, miniturbo coding sequence was amplified with primers ATCCCGCTGCTGAACGCTAAACAGATTCTG and CTTTTCGGCAGACCGCAGACTGATTTCTCC, and inserted by Gibson assembly into the plasmid BirA∗-CL between MluI and XmaI restriction sites [26].

For transfection, late schizonts were isolated on a percoll gradient, mixed with 50 μg of DNA (dissolved in 10 μL TE buffer and 90 μL of Amaxa transfection buffer (90 mM NaPO4, 5 mM KCl, 0.15 mM CaCl2, 50 mM HEPES pH7.3) and electroporated using the Amaxa system (Nucleofector II AAD-1001N Amaxa Biosystems, Germany) nucleofector system (Amaxa), following the U-033 program. After electroporation the parasites were mixed with 300 μL uninfected RBC and 100 μL completed RPMI 1640 medium (0.2% NaHCO3 and 10% human serum) and incubated for 60–90 min at 37°C under rigorous shaking, before being transferred into a 15 × 60 mm Petri dish containing completed RPMI 1640 medium.

#### (DiBio)CUT&Tag nuclei preparation

Parasite cultures were lightly crosslinked with 0.1% formaldehyde (Sigma, F8775), incubating for 2 min at 37°C while shaking. Crosslinking was stopped by addition of glycine to 0.125 M final concentration. Samples were handled on ice from here onwards.

Cells were harvested by 440 g centrifugation for 8 min at 4°C and washed with ice-cold PBS. Centrifugation was repeated and the pellet was washed with PBS supplemented with 1x EDTA-free Protease Inhibitor (Roche, 04693132001). Parasites were extracted by adding saponin (0.05% total concentration) and incubating at room temperature (RT) for 10 min. Nuclei were isolated by carefully transferring the extracted parasite mixture on top of a 0.25 M–0.1 M sucrose gradient in cell lysis buffer (10 mM Tris pH 8, 3 mM MgCl_2_, 0.2% NP-40, 1x EDTA free Protease inhibitor (Roche, 04693132001); 15 mL 0.25 M Sucrose and 17.5 mL 0.1M Sucrose for 50 mL tubes, 4 mL 0.25 M Sucrose and 6 mL 0.1 M Sucrose for 15 mL tubes) and centrifuging for 12 min, 3100 g, 4°C with acceleration and deceleration set to 1 (Eppendorf 5910 Ri, Rotor S-4x400).

Supernatant was removed and nuclei washed in cell lysis buffer (10 min, 3500 g, 4°C; Heraeus Fresco 21). Nuclei were counted in an automatic hemocytometer (BioRad, TC10 Automated Cell Counter) and were directly used for CUT&Tag. Optionally, nuclei were washed with cell lysis buffer containing 20% Glycerol and nuclei pellet was snap frozen in liquid nitrogen prior to storage at −80°C.

#### (DiBio)CUT&Tag parasite isolation

Parasite cultures were harvested by 440 g centrifugation for 8 min at RT and pellet was resuspended in 1 mL of 0.15% Saponin/PBS per 10 mL culture and incubated for 5 min on ice. Samples were vortexed shortly every minute. Samples were washed three times with ice-cold PBS (3500 g centrifugation for 3 min at 4°C; Heraeus Fresco 21). Intact parasites were counted in an automatic hemocytometer and were directly used for CUT&Tag. Optionally, parasites were washed with PBS containing 20% Glycerol and parasite pellets snap frozen in liquid nitrogen prior to storage at −80°C.

#### (DiBio)CUT&Tag reaction

We have adapted the protocol of based on Kaya-Okur et al.^22^ to malaria parasites with changes regarding permeabilization; DNA extraction; Library preparation protocol. Specifically, nuclei or whole parasite were resuspended in CUT&Tag wash buffer (20 mM HEPES pH 7.5, 150 mM NaCl, 0.5 mM Spermidine, 1x EDTA-free protease inhibitor) containing 0.1% Triton X-100 and permeabilized for 10 min on ice. Nuclei/parasites were pelleted by centrifugation (3500 g, 10 min, 4°C) and resuspended in CUT&Tag wash buffer. Concanavalin A beads (Bangs Laboratories, BP531) were activated by resuspending into 10 volumes of Bead Binding Buffer (20 mM HEPES pH 7.5, 10 mM KCl, 1 mM CaCl_2_, 1 mM MnCl_2_), washed once on a magnetic rack and resuspended in the starting volume of bead slurry. Purified nuclei or parasites were bound to 10 μL beads per reaction (or 3 μL beads for low-input samples) by incubating for 10 min rotating at RT. The supernatant was removed and 50 μL antibody buffer (CUT&Tag wash buffer; 2 mM EDTA, 0.1% BSA) with primary antibody (0.25 μL polyclonal rabbit αHP1 ^11^; 0.5 μg αH3K9me3, Abcam 8898 for H3K9me3 CUT&Tag in Figures 1; S1B; 0.5 μg αH3K9me3, Diagenode Cat# C15410193 for H3K9me3 CUT&Tag comparison to literature H3K9me3 ChIP-seq in Figure S1C; 0.5 μL αBiotin (Cell Signaling Technology #D5A7, Cat#:5597; for DiBioCUT&Tag); 0.25μg αGFP (Roche Cat#11814460001); or 0.5 μg normal rabbit IgG (MERCK, Cat#12–370) was added and incubated nutating over night at 4°C. Unbound primary antibody was removed by washing once with 100 μL CUT&Tag wash buffer and samples were then incubated with secondary antibody (1.2 μg guinea pig anti-rabbit antibody, Antibodies-Online ABIN101961; or 0.5 μg goat anti-mouse antibody (EpiCypher 13–0048) in 100 μL CUT&Tag wash buffer, 1:100 dilution for 1h on RT, nutating. The nuclei or parasites were washed on a magnetic stand twice with 100 μL CUT&Tag wash buffer and once with 100 μL CUT&Tag 300 Wash Buffer (20 mM HEPES pH 7.5, 300 mM NaCl, 0.5 mM Spermidine, 1x EDTA-free protease inhibitor). For all following wash steps CUT&Tag 300 wash buffer was used in order to quench potential affinity of protA-Tn5 to accessible chromatin regions. 2.5 μL commercial proteinA/G-Tn5 fusion protein (CUTANA pAG-Tn5 for CUT&Tag, Epicypher, 15–1017) was added in 50 μL CUT&Tag 300 wash buffer and incubated nutating for 1 h. Unbound proteinA/G-Tn5 fusion protein was removed by thrice washing with 100 μL CUT&Tag 300 wash buffer. The supernatant was removed and the nuclei or parasites were resuspended in 200 μL freshly prepared tagmentation buffer (CUT&Tag 300 wash buffer, 10 mM MgCl_2_). To perform tagmentation, samples were incubated in a PCR thermocycler (BioRad, T100) at 37°C for 1h. Tagmentation was stopped and nuclei or parasite lysis was facilitated by addition of 10 μL of 0.5M EDTA pH8, 3 μL of 10% SDS and 1 μL of 50 mg/mL proteinase K. Samples were briefly vortexed and then incubated at 55°C for 1 h. DNA fragments were extracted utilizing the DNA Clean & Concentrator - 5 kit (Zymogen, D4014) following manufactures instructions. DNA was eluted from the column with 26 μL of prewarmed elution buffer and DNA concentrations were assessed with Qubit dsDNA High Sensitivity Assay kit (Invitrogen, Q33231).

#### (DiBio)CUT&Tag library preparation

Maximal 50 ng of extracted DNA from CUT&Tag experiments were amplified with unique combinations of i5 and i7 barcoded primers,^47^ enabling tracing the DNA fragments originating from separate experiments in the sequencing data. PCRs were performed in a total reaction volume of 50 μL using Kapa HiFi polymerase (use non-hotstart version for gap filling; Roche, Cat# 07958838001) in a thermocycler with the following program: 58°C for 5 min, 62°C for 5 min (gap filling), 98°C for 2 min, 12–16 cycles of 98°C for 20 s and 62°C for 10 s, 62°C for 1 min and hold at 4°C. Post-PCR DNA cleanup was performed by adding 50 μL (1x volume) of AMPure XP bead slurry (Beckman Coulter, A63882) and incubating for 10 min at RT, washing twice with 80% EtOH on a magnetic rack, and eluting in 16.5 μL of 10 mM Tris-HCl pH 8 for 5 min at RT. DNA concentrations of libraries were assessed with Qubit dsDNA High Sensitivity Assay kit (Invitrogen, Q33231) and library fragment size distribution was accessed by microfluidic gel electrophoresis (Agilent 2100 Bioanalyser) with the corresponding High Sensitivity DNA Kit (Agilent, 5067-4626). Concentrations from successful libraries were between 0.2 μg/mL to 10 μg/mL, showing hints of a nucleosomal profile with average fragment length from 500 to 700 bp indicative of effective tagmentase activity. Sequencing was performed using an Illumina NextSeq 2000 instrument for 3–10 M reads per CUT&Tag sample; 59bp paired-end reads were generated.

### Quantification and statistical analysis

#### Sequencing data analysis

Sequencing reads were mapped against the reference genome PlasmoDB v26 3D7 using bowtie2 (v2.5.2)^35^^,^^36^ with paired-end mapping for CUT&Tag data and single-end mapping in case of the NF54 HP1 ChIP-seq dataset.^7^ Duplicates in the ChIP-seq datasets were removed with picard (v3.1.0).^37^ Duplicate removal was skipped for CUT&Tag datasets as duplicates may result due to the affinity of Tn5 to certain sequences as well as accessibility of certain regions leading to fragments with the same start and end locations.^48^ Downstream analysis was furthermore not influenced by duplicate removal in pilot analysis of the CUT&Tag datasets.

Reads were filtered by mapping quality ≥ 30 and mitrochondrial as well as apicoplast reads were removed with samtools (v1.18).^38^ BigWig files normalized to read per million per kilobase (RPMK) were created using deeptools (v3.5.4)^39^ for binning of sequencing data into either 500 bp windows for log2 ratio track calculations or 2000 bp windows for correlation analysis. BedGraph files were generated for visualisation purposes on the UCSC genome browser^44^ with bedtools (v2.31.0)^40^ normalized to library size. A detailed & customisable script can be found on our github page https://github.com/bartfai-lab/DiBio-CUTnTag-Analysis or 10.5281/zenodo.15638929.

Log2 ratio tracks were generated by running the multiBigwigSummary command from deeptools (v3.5.4)^39^ using 500 bp bins on both sample and control bigwig. A pseudocount of 1 was added to all values to prevent divisions by 0, and log2 values were calculated in bash and appended to a new bedGraph file for visualisation on UCSC genome browser.^44^

Chromosomal genome-wide overview figures were generated by exporting visualized tracks from the UCSC genome browser, compiling and scaling them to each other in Adobe Illustrator.

When indicated, bedgraph files of replicates were combined into one file with bedtools unionbedg (v2.31.0),^40^ averages per position calculated and a new bedgraph file generated.

#### Signal-to-noise ratio calculations

Signal-to-noise ratios in HP1 CUT&Tag vs. ChIP-seq data were computed using the multiBigwigSummary function from the deeptools package (v3.5.4). Average enrichment over the ATG (±500 bp) was calculated based on heterochromatin genes, as described by Fraschka et al., 2018.^7^ Average enrichment of signal (predicted heterochromatic genes) and noise (euchromatic genes) was calculated and used for final signal-to-noise ratio.

#### Correlation analysis

Average enrichment scores of previously generated bigwig files for read count or log2 ratio tracks were calculated with multiBigwigSummary from the deeptools package (v3.5.4)^39^ on 2000 bp bins. Datasets were imported into R and zero values in the log2 ratio datasets were removed as these are most likely artifacts from adding pseudocounts to non-mappable or not-sequenced genomic regions. If different antibodies were compared, average enrichment scores were filtered for a minimum enrichment score prior to quantitative correlation analysis to remove variations from the background signal in non-background corrected tracks. Scatterplots were generated utilizing the ggplot2 package (v3.4.4),^42^ trendlines and R^2^ values were calculated using the ggpmisc (v0.5.4-1) package in R (v4.3.1).

Heatmaps were generated using the computeMatrix and plotHeatmap commands from the deeptools package (v3.5.4),^39^ using a bed file with the ATG location of all genes as a reference-point and 100 bp bins for the analysis.

#### Modeling/identification of enriched regions

Normal distribution was modeled on 2000 bp binned average enrichment score files previously obtained with the multiBigwigSummary command from the deeptools package (v3.5.4).^39^ Log10 values for the enrichment scores were calculated in order to enable proper visualisation and the normalmixEM function from the mixtools package (v2.0.0.1)^43^ was used to model the normal distribution of the background signal in R. Regions were considered significantly enriched if they showed higher enrichment than the top 0.005% of the background distribution (i.e., 0.5% false discovery rate).

#### Peak identification and expression analysis

Peaks of BDP5 DiBioCUT&Tag were identified using macs2 (v2.2.9.1)^41^ using the IgG CUT&Tag background track as a control and a q-value of 0.005. Any peaks falling in telomeric regions before the first annotated var gene were removed with bedtools intersect (v2.31.0).^40^ For each peak, the closest ATG was identified using bedtools closest (v2.31.0)^40^ and peak profiles were generated using the deeptools package (v3.5.4).^39^ For expression analysis, peaks were intersected with the 5′ end of genes (−1000bp from ATG and 500bp into the coding body). RNA expression values of affected genes at different timepoints were obtained from Toenhake et al., 2018 RNAseq dataset.^49^ Mean expression values of all affected genes were calculated and plotted using the ggplot2 package (v3.4.4).^42^

## Supplemental information

Document S1. Figures S1–S4

Document S2. Article plus supplemental information
