# Supplementary material for: CUT&Tag and DiBioCUT&Tag enable investigation of the AT-rich epigenome of Plasmodium falciparum from low-input samples
Source: Cell Rep Methods. 2025 Jul 16;5(8):101110. doi: 10.1016/j.crmeth.2025.101110 (PMC12461585; doi:10.1016/j.crmeth.2025.101110)
Supplement: Document S1. Figures S1–S4 [file mmc1.pdf]

Cell Reports Methods, Volume *5*

Supplemental information

CUT&Tag and DiBioCUT&Tag enable investigation of the AT-rich epigenome of *Plasmodium falciparum* from low-input samples

Jonas Gockel, Gala Ramón-Zamorano, Jessica Kimmel, Tobias Spielmann, and Richárd Bártfai

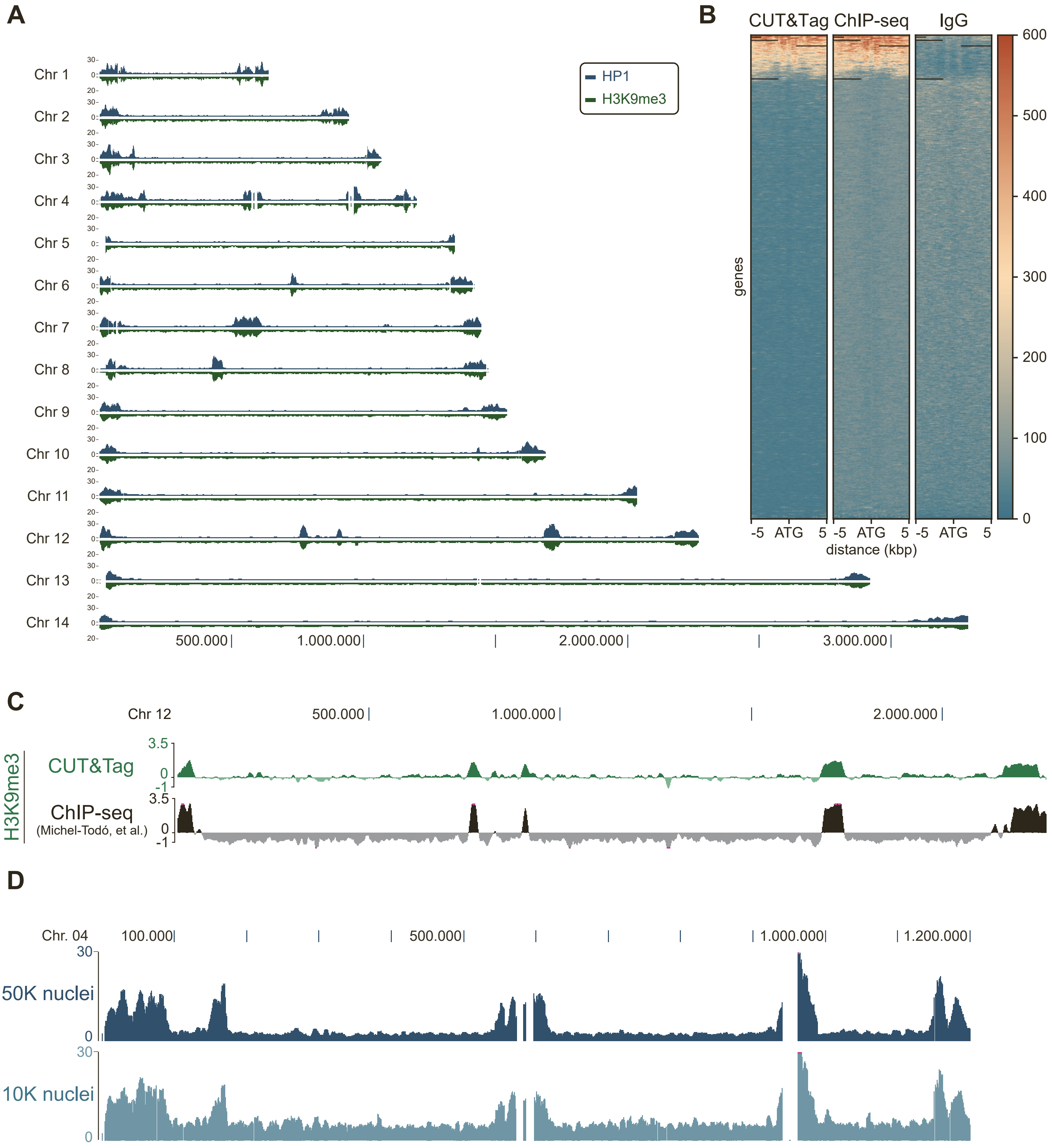

Supplementary Figure 1: (low-input) CUT&Tag reproducibly profiles heterochromatin in *P. falciparum*. Related to Figures 1 and 2.

A) Genome-wide view of read-occupancy profiles for HP1 (blue) and H3K9me3 (green).

B) Heatmap depicting assigned reads in raw HP1 CUT&Tag, HP1 ChIP-seq and IgG CUT&Tag in relation to the ATG of all genes.

C) Log2 ratio tracks of H3K9me3 CUT&Tag and ChIP-seq (Diagenode H3K9me3, RRID:AB_2616044). ChIP-seq data was generated by Michel-Todó et. al., 2023.

D) Read occupancy profiles of HP1 CUT&Tag performed on 50.000 nuclei (dark blue) and 10.000 nuclei (light blue).

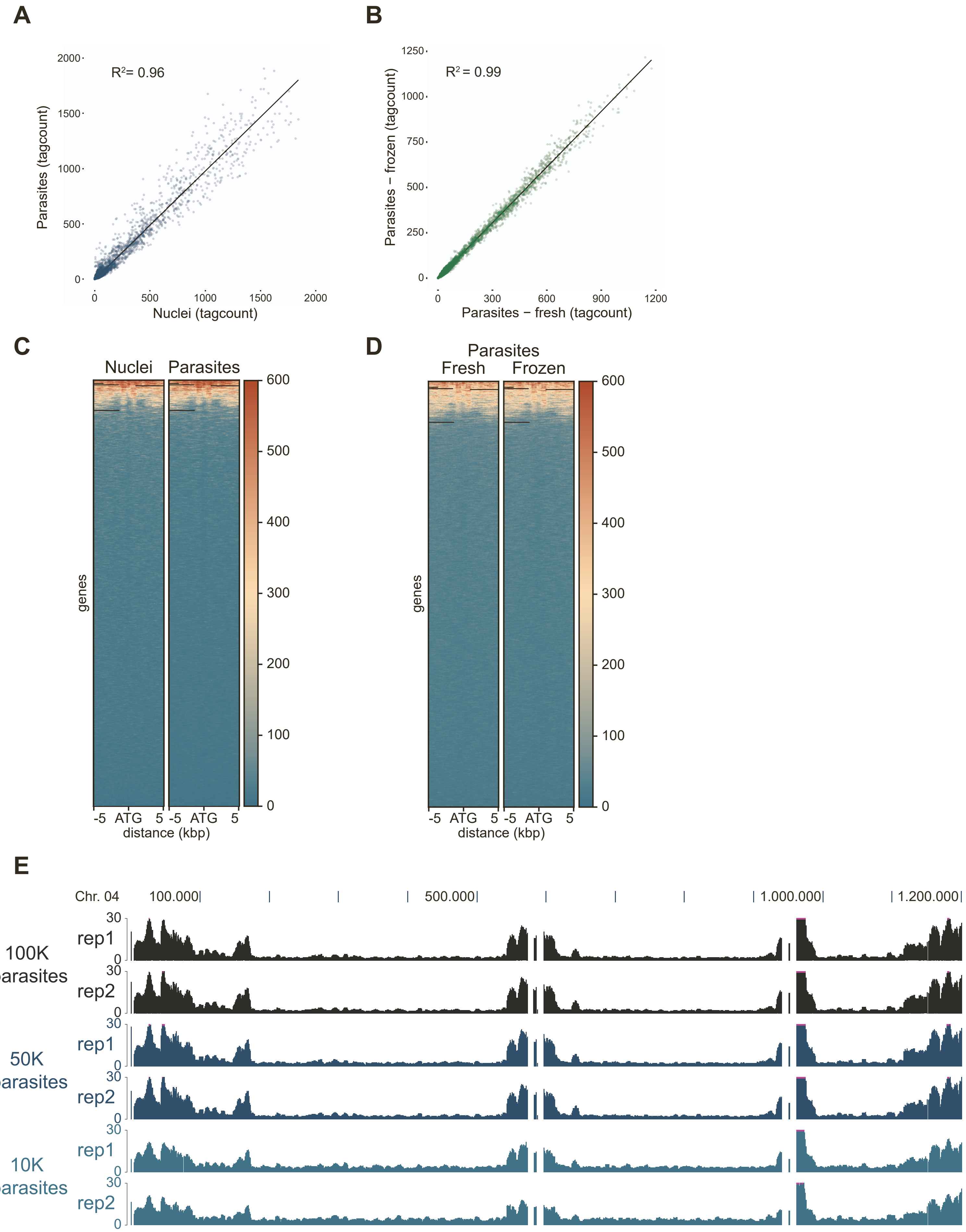

Supplementary Figure 2: CUT&Tag on (frozen) crude parasite isolates. Related to Figure 3.

A) Scatter plot displaying correlation between isolated parasites and isolated nuclei as input material for CUT&Tag in 2000bp windows genome-wide.

B) Scatter plot displaying correlation between frozen isolated and freshly isolated parasites as input material for CUT&Tag in 2000bp windows genome-wide.

C/D) Comparison of reads in the vicinity of ATGs of all genes between CUT&Tag using nuclei or crude parasite isolates (C) or fresh and frozen parasite isolates (D) as input.

E) Read occupancy profiles of HP1 CUT&Tag performed on 100.000 (dark blue), 50.000 (blue) and 10.000 (light blue) parasite isolates.

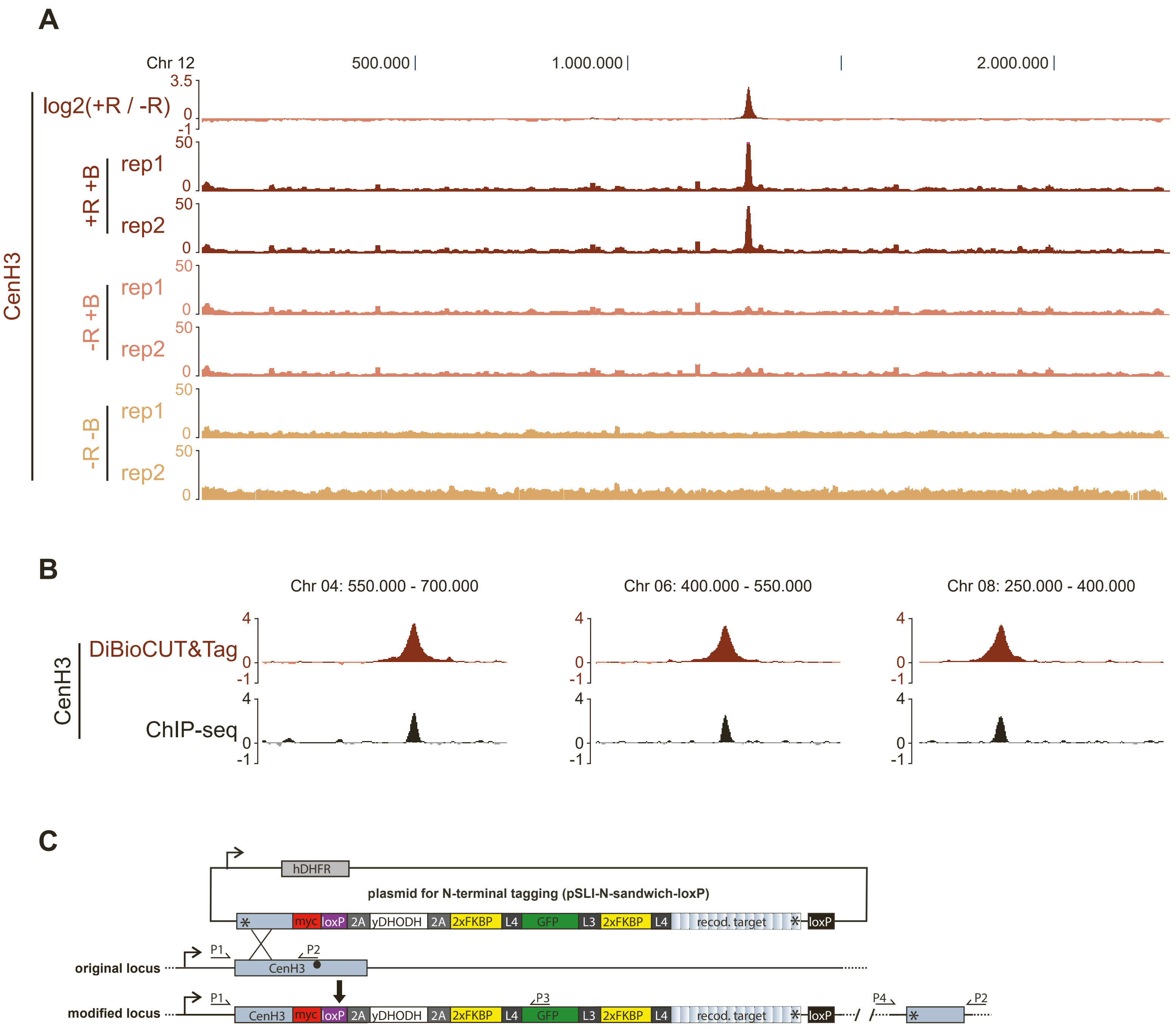

P1	CENH3_int_fw	TCGACAAATTGTTCACCTAGC
P2	CENH3_int_rv	ATCCTGCGTACACAAATTGC
P3	GFP 272 as	CCTTCGGGCATGGCACTC
P4	pARLsense 55	GGAATTGTGAGCGGATAACAATTTCACACAGG

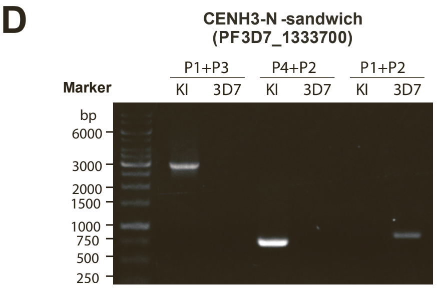

Supplementary Figure 3: DiBioCUT&Tag of CenH3 demarkates centromeres / CenH3 line validation. Related to Figure 4.
A) Read-occupancy profiles of DiBioCUT&Tag (2 replicates) on CenH3 cultured in different Rapalog (R) or Biotin (B) conditions.
B) Zoom in to centromere regions for Chromosome 4, 6 and 8, comparing log2 ratio background corrected DiBioCUT&Tag and ChIP-seq tracks.
C) Diagram of the pSLI-N-sandwich-loxP plasmid containing the homology region for the recombination in the original CenH3 genomic locus. The primers used for validation of the modified locus are indicated.
D) PCR amplification of the modified and original locus sequences from gDNA of knock-in (KI) and wildtype (3D7) lines, respectively.

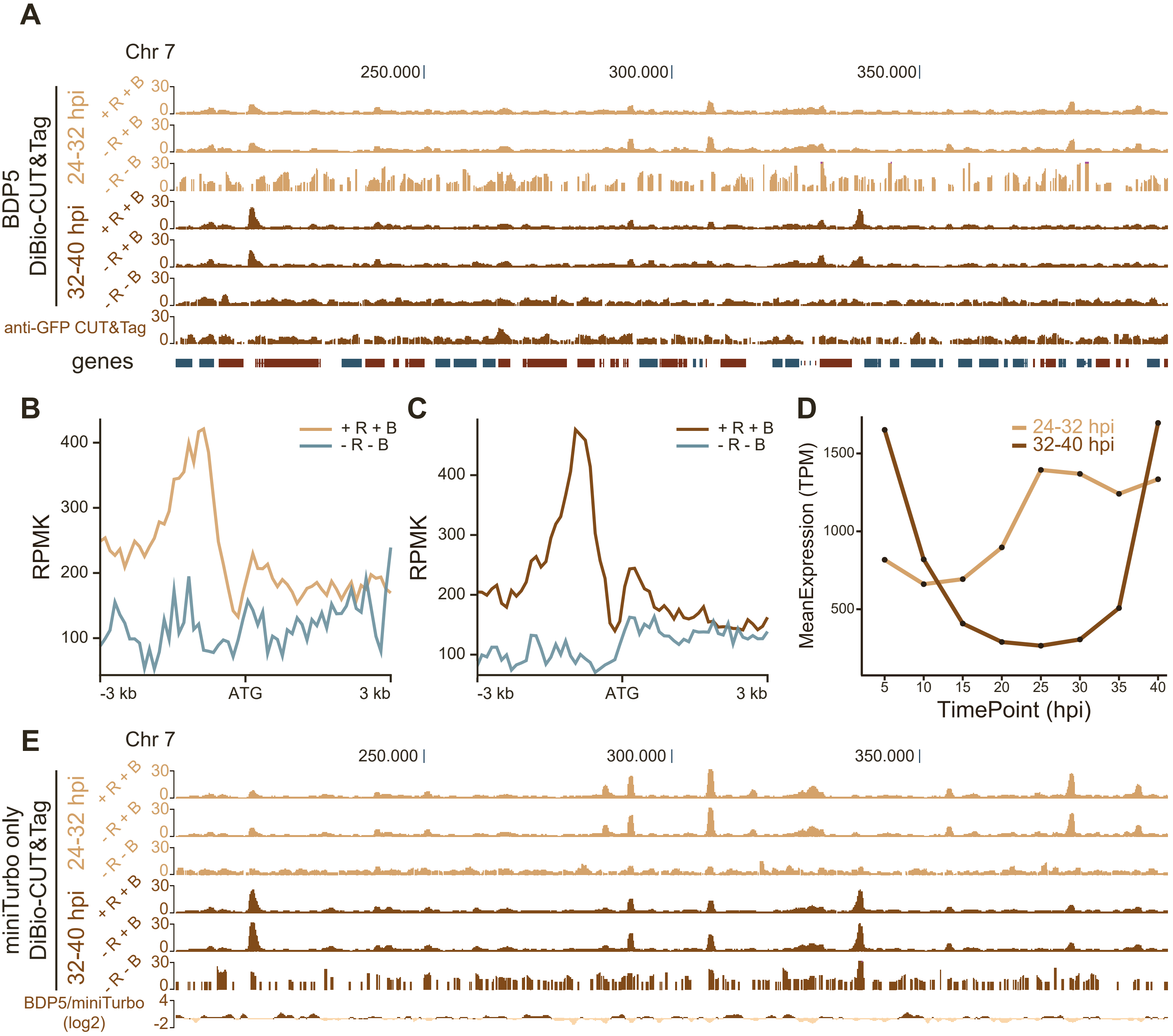

Supplementary Figure 4: DiBioCUT&Tag background signal coincides with regulatory regions of active genes. Related to Figure 4.

A) Read-occupancy profiles of DiBioCUT&Tag on BDP5 cultured in different Rapalog (R) or Biotin (B) conditions at 24-32 hpi (orange) and 32-40 hpi (brown) as well as anti-GFP CUT&Tag on BDP5 in the same parasite line. DiBioCUT&Tag signal is average of two replicates.

B) Peak profiles of 24-32 hpi BDP5 DiBioCUT&Tag (orange) and control (blue; no biotin and no rapalog) in relation to the ATG.

C) Peak profiles of 32-40 hpi BDP5 DiBioCUT&Tag (brown) and control (blue; no biotin and no rapalog) in relation to the ATG.

D) Mean expression of genes (transcripts per kilobase million) bound by BDP5 up to 1000 bp before and 500 bp after ATG at different times of the life cycle (24-32 and 32-40 hpi) as defined by Toenhake et al 2018 [37].

E) Read-occupancy profiles of DiBioCUT&Tag in a miniTurbo only expressing parasite strain in different Rapalog (R) or Biotin (B) conditions at 24-32 hpi (orange) and 32-40 hpi (brown) as well as a log2 ratio track background correcting BDP5 DiBio-CUT&Tag with the miniTurbo only control. All tracks depicted are averages from two replicates.
